# Mitomycin, 5-fluorouracil, leflunomide, and mycophenolic acid directly promote hepatitis B virus replication and expression in vitro

**DOI:** 10.1186/s12985-020-01339-5

**Published:** 2020-07-01

**Authors:** Jie Ruan, Shuo Sun, Xin Cheng, Pengyu Han, Yinge Zhang, Dianxing Sun

**Affiliations:** 1 The Liver Disease Diagnosis and Treatment Center of PLA, Bethune International Peace Hospital, Zhongshanxi street, Shijiazhuang, 050082 Hebei Province China; 2 Department of Infection and Liver Disease, Shaanxi University of Chinese Medicine, Xianyang, 712000 Shaanxi Province China

**Keywords:** Reactivation of hepatitis B virus; Mitomycin, 5-fluorouracil, Leflunomide, Mycophenolic acid, Lamivudine

## Abstract

**Background:**

Reactivation of hepatitis B virus is a common complication that occurs in patients with hepatitis B virus (HBV) infection who have received cytotoxic chemotherapy or immunosuppressive therapy. This clinical phenomenon not only occurs in overt HBV infection patients but also occurs in patients with resolved HBV infection. Previous research has confirmed that epirubicin and dexamethasone can stimulate HBV replication and expression directly rather than indirectly through immunosuppression. Mitomycin and 5-fluorouracil are currently used as cytotoxic chemotherapy drugs for cancer patients. Leflunomide and mycophenolic acid are regarded as immunosuppressants for autoimmune diseases, and numerous clinical studies have reported that these drugs can reactivate HBV replication. In this study, we aimed to investigate whether mitomycin, 5-fluorouracil, leflunomide and mycophenolic acid induce HBV reactivation directly rather than indirectly through immunosuppression.

**Methods:**

To observe the effect of mitomycin, 5-fluorouracil, leflunomide and mycophenolic acid on HBV replication and expression, we employed HepG2.2.15 and HBV-NLuc-35 cells as a cell model. Next, by native agarose gel electrophoresis (NAGE), quantitative PCR (qPCR), luciferase assay and HBV e antigen (HBeAg) enzyme-linked immunosorbent assay (ELISA) we detected changes in HBV replication and expression induced by these drugs. We also investigated whether lamivudine could inhibit the observed phenotype. SPSS 18.0 software was employed for statistical analysis, One-way ANOVA was used to compare multiple groups.

**Results:**

Expression of HBV capsids and HBeAg in HepG2.2.15 cells was increased by increasing concentration of mitomycin, 5-fluorouracil, leflunomide, and mycophenolic acid. This phenomenon was also demonstrated in HBV-NLuc-35 cells, and the expression of capsids and luciferase activity increased in the same concentration-dependent manner. Replication levels of intracellular capsid DNA and extracellular HBV DNA in HepG2.2.15 cells gradually increased in a dose-dependent manner. In addition, although epirubicin, mitomycin, 5-fluorouracil, dexamethasone, leflunomide and mycophenolic acid enhanced HBV replication, lamivudine inhibited this process.

**Conclusion:**

Our study confirmed that mitomycin, 5-fluorouracil, leflunomide and mycophenolic acid directly upregulated HBV replication and expression in vitro. This effect was investigated not only in HepG2.2.15 cells but also in the HBV-NLuc-35 replication system. Moreover, this effect could be prevented by nucleoside analogs, such as lamivudine (LAM). Thus, for patients with HBV infection, prophylactic antiviral therapy is necessary before receiving cytotoxic chemotherapy or immunosuppressive therapy.

## Background

Reactivation of hepatitis B is a common complication that occurs in patients with HBV infection who receive cytotoxic chemotherapy or immunosuppressive therapy [1–4]. The first case of hepatitis B reactivation was described in 1975 [5, 6], and the studies reported that the patient was HBV surface antigen positive and received cytotoxic chemotherapy for cancer. The sign of hepatitis B reactivation is the reoccurrence of HBV DNA in the serum of patients with cured or inactive infection, often accompanied by inflammation activity in the liver and could occur spontaneously but mostly occurs during or after cytotoxic chemotherapy or immunosuppressive therapy, which could lead to acute hepatitis, liver failure, or even death [7, 8]. Nevertheless, most cases are not clinically significant or are not diagnosed until the infection has developed into active hepatitis, which leads to the interruption of cytotoxic chemotherapy or immunosuppressive therapy and a poor prognosis [9, 10].

HBV reactivation starts with an enhancement of HBV replication and occurs shortly after cytotoxic chemotherapy or immunosuppressive therapy [11, 12], and the mechanism of this phenomenon has not been determined thus far. Previous research had confirmed that dexamethasone [13] and epirubicin [14, 15] can increase HBV replication and expression in vitro, which suggests that dexamethasone and epirubicin could stimulate viral replication and expression directly rather than indirectly through immunosuppression. Numerous clinical studies have reported that mitomycin [1], 5-fluorouracil [1, 16], leflunomide [17], and mycophenolic acid [18] treatment can reactivate HBV replication. In this study, we aimed to investigate whether mitomycin, 5-fluorouracil, leflunomide and mycophenolic acid induce HBV replication in the same manner; thus, we employed HepG2.2.15 cells as a cell model [19]. Moreover, we also investigated the effect of these cytotoxic chemotherapy drugs and immunosuppressants on the new HBV replication system HBV-NLuc-35 cells [20], which are generated by transfecting the pTRE-sNLuc vector into HepG2 TA-7 cells [21, 22] and could stably secrete secNluc recombinant HBV particles, detected the level of secNLuc expression to evaluate HBV replication and expression.

## Methods

### Drugs, cell culture and toxicity test

Epirubicin, mitomycin (Hisun, China), 5-fluorouracil (Shanghai Xudong Haipu Pharmaceutical Co. Ltd., China), dexamethasone (Solarbio, China), lamivudine (Xinjialin Biotech Ltd.), were diluted by double distilled water and stored at 4 °C; leflunomide (Sangon Biotech, China), mycophenolic acid (BioDee, China), were diluted in dimethyl sulfoxide and stored at 4 °C; and HepG2.2.15 and HBV-NLuc-35 cells were cultured as previously described [21]. For detecting the cell proliferation toxicity of drugs, HepG2.2.15 cells were treated with increasing concentrations of epirubicin (0, 0.1, 0.5, 1, 2.5 and 5 μM), mitomycin (0, 0.5, 1, 2.5, 5 and 10 μM), 5-fluorouracil (0, 5, 10, 50, 100 and 150 μM) for 16 h, and increasing concentrations of dexamethasone (0, 0.1, 1, 10, 50 and 100 μM), leflunomide (0, 10, 50, 100, 200 and 300 μM), and mycophenolic acid (0, 3, 15, 30, 75 and 150 μM) for 5 days. In addition, the cell proliferation toxicity test of HBV-Nluc-35 cells under different concentrations of Mitomycin treatment was the same as described for HepG2.2.15 cells. Next, the cell proliferation toxicity test was performed by using the CCK-8 reagent as recommended by the manufacturer (Dojindo, Japan).

### Native agarose gel electrophoresis (NAGE), quantitative PCR (qPCR)

Cytoplasmic intact capsids were detected by anti-HBV core protein antibody after native agarose gel electrophoresis (NAGE) as previously described [23]. Moreover, capsid DNA was detected by molecular hybridization with the α-^32^P-labeled HBV DNA probe [24]. HBV DNA copies in the supernatant were detected by a commercial HBV DNA kit (Kehua, Shanghai, China) on a SLAN™ Real-Time PCR system (Hongshi, Shanghai, China).

### Luciferase activity, HBeAg ELISA

Luciferase activity of the culture supernatant was detected by the Nano-Glo™ luciferase assay reagent as recommended by the manufacturer (Promega). HBeAg in culture supernatants was assayed by commercial ELISA kits (Chemclin Biotech Co. Ltd., Beijing, China).

### Drug susceptibility assays

HepG2.2.15 and HBV-NLuc-35 cells were seeded in 96-well plates at a density of 2 × 10^4^ cells/well. For observing the effect of the drugs, the cells were incubated with increasing concentrations of epirubicin, mitomycin, and 5-fluorouracil for 16 h and treated with increasing concentrations of dexamethasone, leflunomide, and mycophenolic acid for 5 days. Intact capsids and capsid DNA of cytoplasmic lysates were analyzed by NAGE. Luciferase, HBeAg and HBV DNA in the cell supernatant were assessed by the Nano-Glo™ luciferase assay reagent, ELISA, and qPCR, respectively. For observing lamivudine sensitivity, HepG2.2.15 cells were treated with increasing concentrations of lamivudine (0, 1, 10, 20 and 50 μM) for 24 h and then treated with the following drugs for 16 h: 1 μM epirubicin, 1 μM mitomycin, or 100 μM 5-fluorouracil or were similarly treated with the following drugs for 5 days: 10 μM dexamethasone, 50 μM leflunomide, or 30 μM mycophenolic acid. Then, cell media was replaced with drug-free culture media, followed by incubation with increasing concentrations of lamivudine for 24 h. Subsequently, the cytoplasmic lysates were analyzed for intracellular capsid DNA by NAGE, and HBV DNA in the culture supernatant was detected by qPCR.

### Statistical analysis

SPSS 18.0 software was employed for statistical analysis. We calculated the median lethal dose (LD_50_) for drugs with modules in the SPSS 18.0 package, and the 95% CI of LD_50_ was obtained at the same time. One-way ANOVA was used to compare multiple groups, and all data were expressed as the mean with standard deviation (SD). *P* < 0.05 was regarded as statistically significant.

## Results

### Cell proliferation toxicity test

**Fig. 1:**
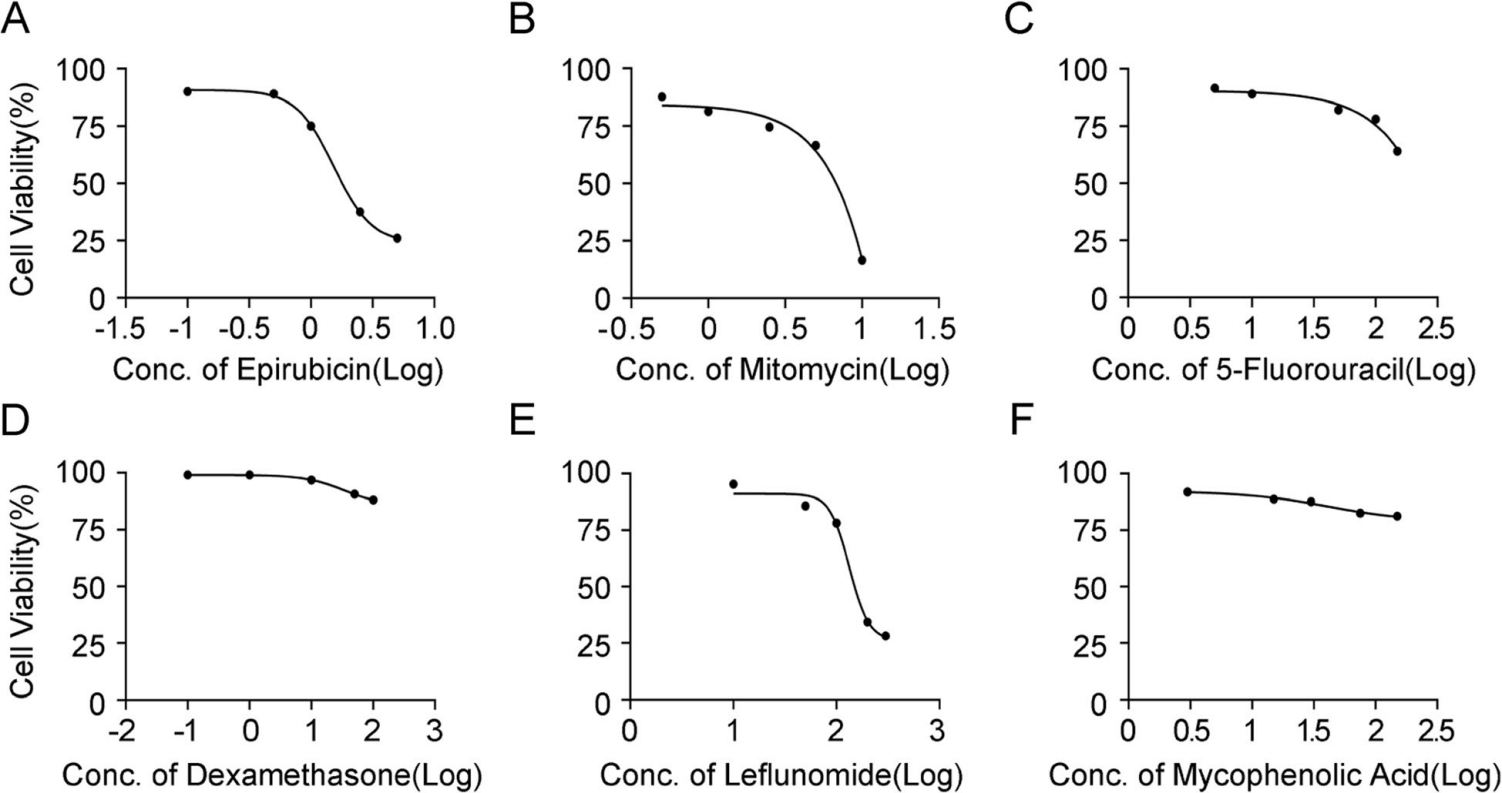
Cell proliferation toxicity test of HepG2.2.15 cells treated with the different drugs. HepG2.2.15 cells were treated with increasing concentrations of epirubicin (**a**), mitomycin (**b**), and 5-fluorouracil (**c**) for 16 h and with increasing concentrations of dexamethasone (**d**), leflunomide (**e**), and mycophenolic acid (**f**) for 5 days. Cell proliferation toxicity was measured using CCK-8 reagent as recommended by the manufacturer (Dojindo)

We used the CCK-8 reagent to detect the cell proliferation toxicity of drugs in HepG2.2.15 cells which treated with different concentrations of epirubicin, mitomycin, 5-fluorouracil, dexamethasone, leflunomide, and mycophenolic acid at the indicated times. The results showed that the proliferation activity of HepG2.2.15 cells was reduced below 50% with concentrations of epirubicin up to 2.5 μM (Fig. 1a). The LD_50_ was 2.085 μM, and the 95% CI of the LD_50_ was 1.3–3.394 μM. The same result was observed for mitomycin up to 10 μM (Fig. 1b), leflunomide up to 200 μM (Fig. 1e). The LD_50_ of these drugs were 5.405 μM and 172.2 μM, and the 95% CIs of the LD_50_ were 2.507–12.43 μM and 89.99–341.7 μM, respectively. Regarding 5-fluorouracil (Fig. 1c), dexamethasone (Fig. 1d) and mycophenolic acid (Fig. 1f), when concentrations up to 150 μM, 100 μM and 150 μM, respectively, were used, the proliferation activity of HepG2.2.15 cells was decreased but above 50%. The LD_50_ of these drugs were 276 μM, 641.9 μM and 459.1 μM, and the 95% CIs of the LD_50_ were (176.4–480.5) μM, (471.0–964.4) μM and (219.3–1877) μM, respectively. Hence, for further study, we chose the following lower toxicity concentrations of the drugs: epirubicin (0, 0.01, 0.05, 0.1, 0.5 and 1 μM), mitomycin (0, 0.1, 0.5, 1, 2.5 and 5 μM), 5-fluorouracil (0, 1, 5, 10, 50 and 100 μM), dexamethasone (0, 0.1, 1, 10, 50 and 100 μM), leflunomide (0, 1, 5, 10, 50 and 100 μM), and mycophenolic acid (0, 3, 15, 30, 75 and 150 μM).

### Concentration-dependent expression of HBV antigens in HepG2.2.15 cells stimulated by epirubicin, mitomycin, 5-fluorouracil, dexamethasone, leflunomide, and mycophenolic acid

**Fig. 2:**
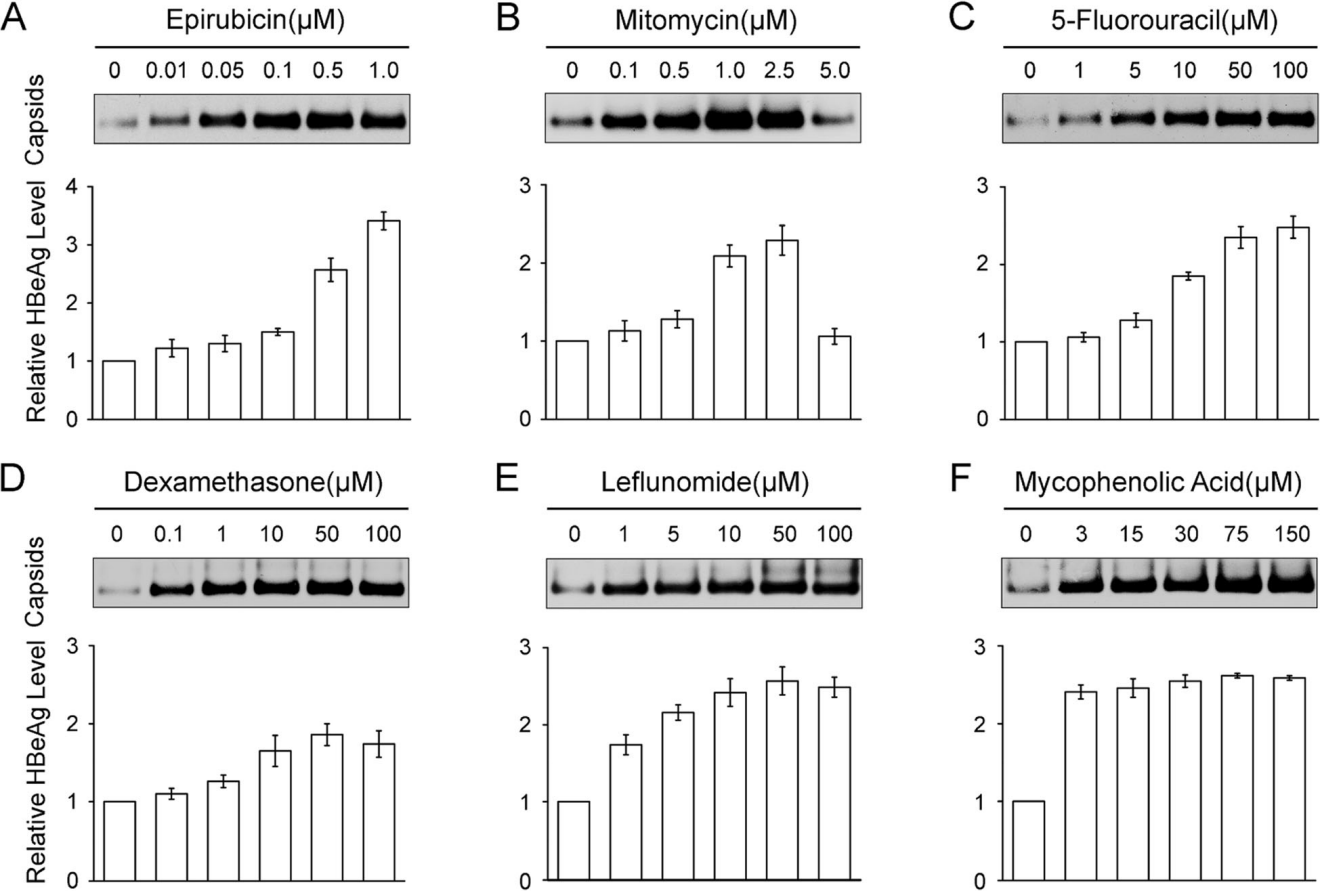
Expression of HBV capsids and HBeAg in HepG2.2.15 cells is affected by epirubicin, mitomycin, 5-fluorouracil, dexamethasone, leflunomide, and mycophenolic acid. HepG2.2.15 cells were treated with increasing concentrations of cytotoxic chemotherapy drugs, epirubicin (**a**), mitomycin (**b**), and 5-fluorouracil (**c**), for 16 h, and increasing concentrations of immunosuppressants, dexamethasone (**d**), leflunomide (**e**), and mycophenolic acid (**f**), for 5 days. Intact intracellular capsids were detected by immunoblotting with an anti-HBV core protein antibody after NAGE (upper). Levels of secreted HBeAg were determined by ELISA (bottom); values are the mean of three isodose treatments, error bars indicate SD, and differences between groups are statistically significant (*p* < 0.05)

To test if HBV antigen expression was affected by epirubicin, mitomycin, 5-fluorouracil, dexamethasone, leflunomide, and mycophenolic acid, we employed HepG2.2.15 cells as a cell model, and treated the cells with various doses of drugs. According to the cell proliferation toxicity test results, we selected a safe dose and exposed HepG2.2.15 cells to increasing concentrations of drugs to observe the effect. Following treatment with increasing concentrations of drugs, the formation of HBV capsids in inside of cells was upregulated in a dose-dependent manner. NAGE results showed that capsid signals from HepG2.2.15 cells treated with gradually increasing concentrations, increased with epirubicin up to 1 μM (Fig. 2a, upper), mitomycin up to 2.5 μM (Fig. 2b, upper), 5-fluorouracil up to 100 μM (Fig. 2c, upper), dexamethasone up to 50 μM (Fig. 2d, upper), leflunomide up to 50 μM (Fig. 2e, upper), and mycophenolic acid up to 150 μM (Fig. 2f, upper). The comparable HBeAg ELISA results revealed that the level of HBeAg in the culture supernatant was over 3 times that of the untreated control when the concentration of epirubicin increased to 1 μM (Fig. 2a, bottom). In addition, when mitomycin increased to 2.5 μM (Fig. 2b, bottom), 5-fluorouracil to 100 μM (Fig. 2c, bottom), dexamethasone to 50 μM (Fig. 2d, bottom), leflunomide to 50 μM (Fig. 2e, bottom), and mycophenolic acid to 150 μM (Fig. 2f, bottom), the level of HBeAg was over 2 times that of the untreated control. Notably, when the concentration of mitomycin was up to 5 μM, both the expression level of HBV capsids and HBeAg declined because HepG2.2.15 cells were more sensitive to toxicity at this concentration. Altogether, expression of HBV capsids and HBeAg increased in HepG2.2.15 cells stimulated with increasing concentrations of epirubicin, mitomycin, 5-fluorouracil, dexamethasone, leflunomide, and mycophenolic acid.

### Concentration-dependent expression of HBV genes in HBV-NLuc-35 cells induced by epirubicin, mitomycin, 5-fluorouracil, dexamethasone, leflunomide, and mycophenolic acid

**Fig. 3:**
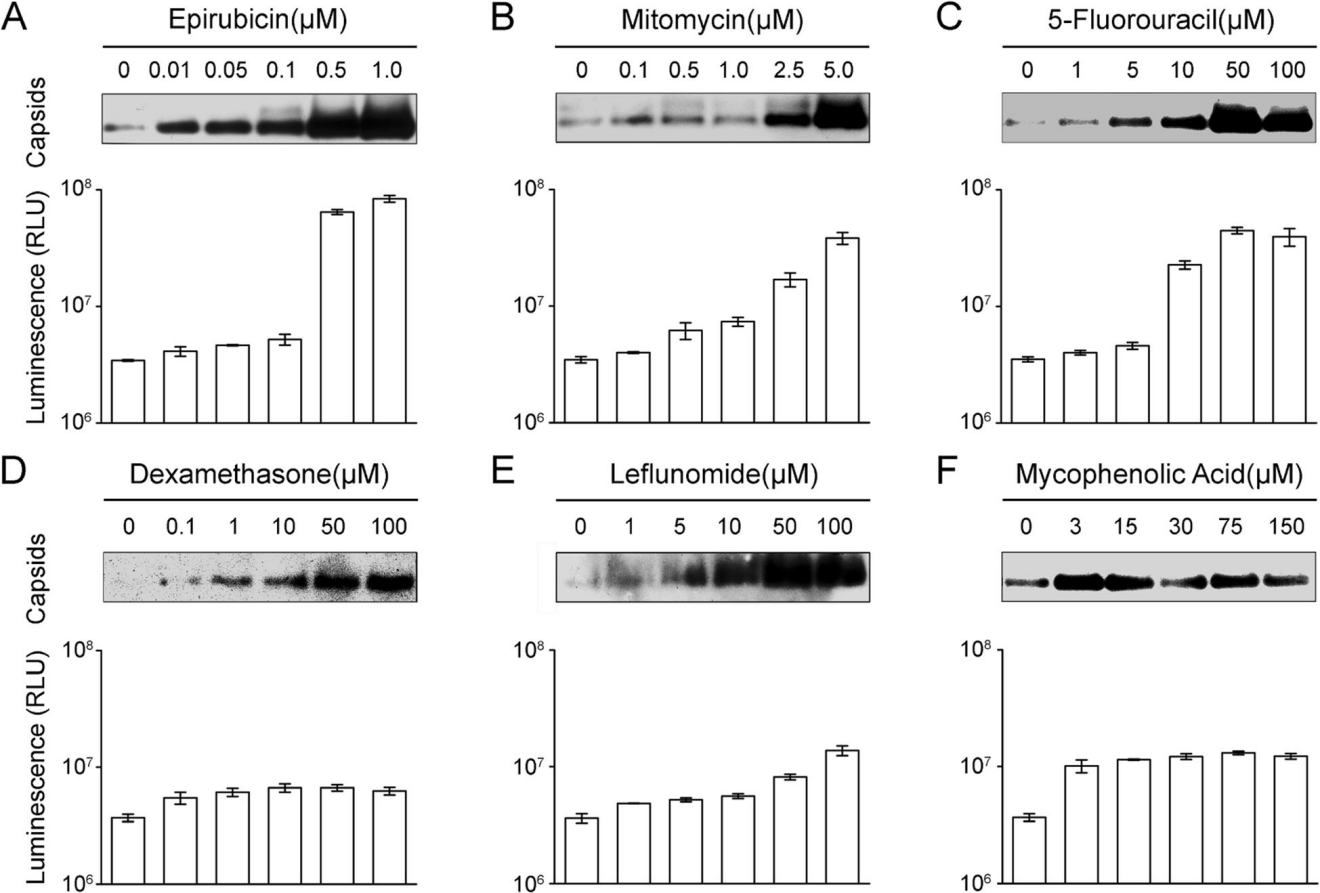
Expression of HBV genes in HBV-NLuc-35 cells is affected by epirubicin, mitomycin, 5-fluorouracil, dexamethasone, leflunomide, and mycophenolic acid. HBV-NLuc-35 cells were exposed to increasing concentrations of cytotoxic chemotherapy drugs, epirubicin (**a**), mitomycin (**b**), and 5-fluorouracil (**c**), for 16 h, and increasing concentrations of immunosuppressants, dexamethasone (**d**), leflunomide (**e**), and mycophenolic acid (**f**), for 5 days. Intact intracellular capsids were detected by immunoblotting with an anti-HBV core protein antibody after NAGE (upper). Luciferase activity in supernatant was determined by the Nano-Glo™ luciferase assay reagent (bottom). Values are the mean of three isodose treatments. Error bars indicate SD. Differences among each concentration are statistically significant (*p* < 0.05)

To investigate whether this effect was similar in other HBV replication and expression cell lines, we employed HBV-NLuc-35 cells. These cells were exposed to increasing doses of epirubicin, mitomycin, 5-fluorouracil, dexamethasone, leflunomide, and mycophenolic acid in the same as with the HepG2.2.15 cells. The formation of HBV capsids in inside of cells was detected by NAGE, and the results showed that capsids signals from HBV-NLuc-35 cells also gradually increased with increasing concentrations (Fig. 3, upper). However, one difference was that when the concentration of Mitomycin increased to 5 μM, the capsid signals from HBV-NLuc-35 cells was stronger compared with the weaker signals from HepG2.2.15 cells, the reason was that HBV-NLuc-35 cells were more tolerant than HepG2.2.15 cells at this concentration (Supplemental figure). Moreover, we also detected the luciferase level in the cell supernatant, and an identical result was observed (Fig. 3, bottom). The luciferase level was up to 10^8^ RLU when the concentration of epirubicin increased to 1 μM compared with the lower 10^6^ RLU in the negative control group (Fig. 3a, bottom). In addition, when mitomycin was up to 5 μM (Fig. 3b, bottom), 5-fluorouracil to 50 μM (Fig. 3c, bottom), leflunomide to 50 μM (Fig. 3e, bottom), and mycophenolic acid to 3 μM (Fig. 3f, bottom), the luciferase level increased to over 10^7^ RLU compared to the baseline 10^6^ RLU. The effect of dexamethasone was relatively weaker, and the luciferase level was under 10^7^ RLU but over baseline 10^6^ RLU when dexamethasone was up to 50 μM (Fig. 3d, bottom). These data demonstrated that treatment with epirubicin, mitomycin, 5-fluorouracil, dexamethasone, leflunomide, and mycophenolic acid promoted HBV gene expression not only in HepG2.2.15 cells but also in another HBV replication and expression cell line, HBV-NLuc-35 cells.

### Epirubicin, mitomycin, 5-fluorouracil, dexamethasone, leflunomide, and mycophenolic acid dose-dependently enhance HBV DNA replication in HepG2.2.15 cells

**Fig. 4:**
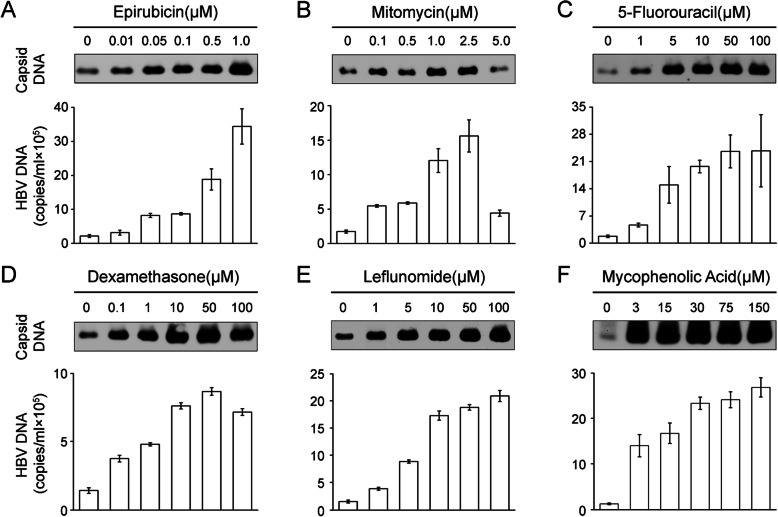
Replication of HBV genes in HepG2.2.15 cells stimulated by epirubicin, mitomycin, 5-fluorouracil, dexamethasone, leflunomide, and mycophenolic acid. HepG2.2.15 cells were exposed to increasing concentrations of cytotoxic chemotherapy drugs, epirubicin (**a**), mitomycin (**b**), and 5-fluorouracil (**c**), for 16 h, and increasing concentrations of immunosuppressants, dexamethasone (D), leflunomide (**e**), and mycophenolic acid (**f**), for 5 days. Intracellular capsid DNA was detected by molecular hybridization with the α-^32^P-labeled HBV DNA probe after NAGE (top). The number of extracellular HBV DNA copies was measured by qPCR (bottom), and the mean values from three isodose treatments, including SD, were statistically significant (*p* < 0.05)

The effect of epirubicin, mitomycin, 5-fluorouracil, dexamethasone, leflunomide and mycophenolic acid on HBV replication was examined after treatment with increasing doses of these drugs in HepG2.2.15 cells. The intracellular capsid DNA was analyzed by Native Southern blotting using radioactively labeled HBV DNA as a probe. The results revealed that capsid DNA signals were gradually enhanced with increasing doses and were stronger at 1 μM epirubicin (Fig. 4a, upper), 2.5 μM mitomycin (Fig. 4b, upper), 100 μM 5-fluorouracil (Fig. 4c, upper), 50 μM dexamethasone (Fig. 4d, upper), 100 μM leflunomide (Fig. 4e, upper) and 150 μM mycophenolic acid (Fig. 4f, upper). For comparison, extracellular HBV DNA was detected by qPCR, revealing that HBV DNA copies in the supernatant gradually increased in a dose-dependent manner (Fig. 4, bottom). When the concentration of epirubicin was up to 1 μM, the cells produced as many as 3 × 10^6^ copies/ml of virions compared with 10^5^ copies/ml in the untreated cells (Fig. 4a, bottom). A similar result was observed with mitomycin up to 2.5 μM (Fig. 4b, bottom), 5-fluorouracil up to 100 μM (Fig. 4c, bottom), leflunomide up to 100 μM (Fig. 4e, bottom) and mycophenolic acid up to 150 μM (Fig. 4f, bottom). In addition, the effect of dexamethasone was weaker which only produced approximately 8 × 10^5^ copies/ml of virions with concentrations up to 50 μM (Fig. 4d, bottom). Notably, when the concentration of mitomycin was up to 5 μM, the replication level of both intracellular and extracellular HBV DNA significantly declined. A reasonable explanation was that the cell proliferation toxicity of 5 μM mitomycin was strong enough to affect the growth of HepG2.2.15 cells. These data confirmed that epirubicin, mitomycin, 5-fluorouracil, leflunomide, mycophenolic acid, and dexamethasone could stimulate HBV replication in vitro.

### Lamivudine inhibits the reactivation of HBV replication induced by epirubicin, mitomycin, 5-fluorouracil, leflunomide, mycophenolic acid and dexamethasone

**Fig. 5:**
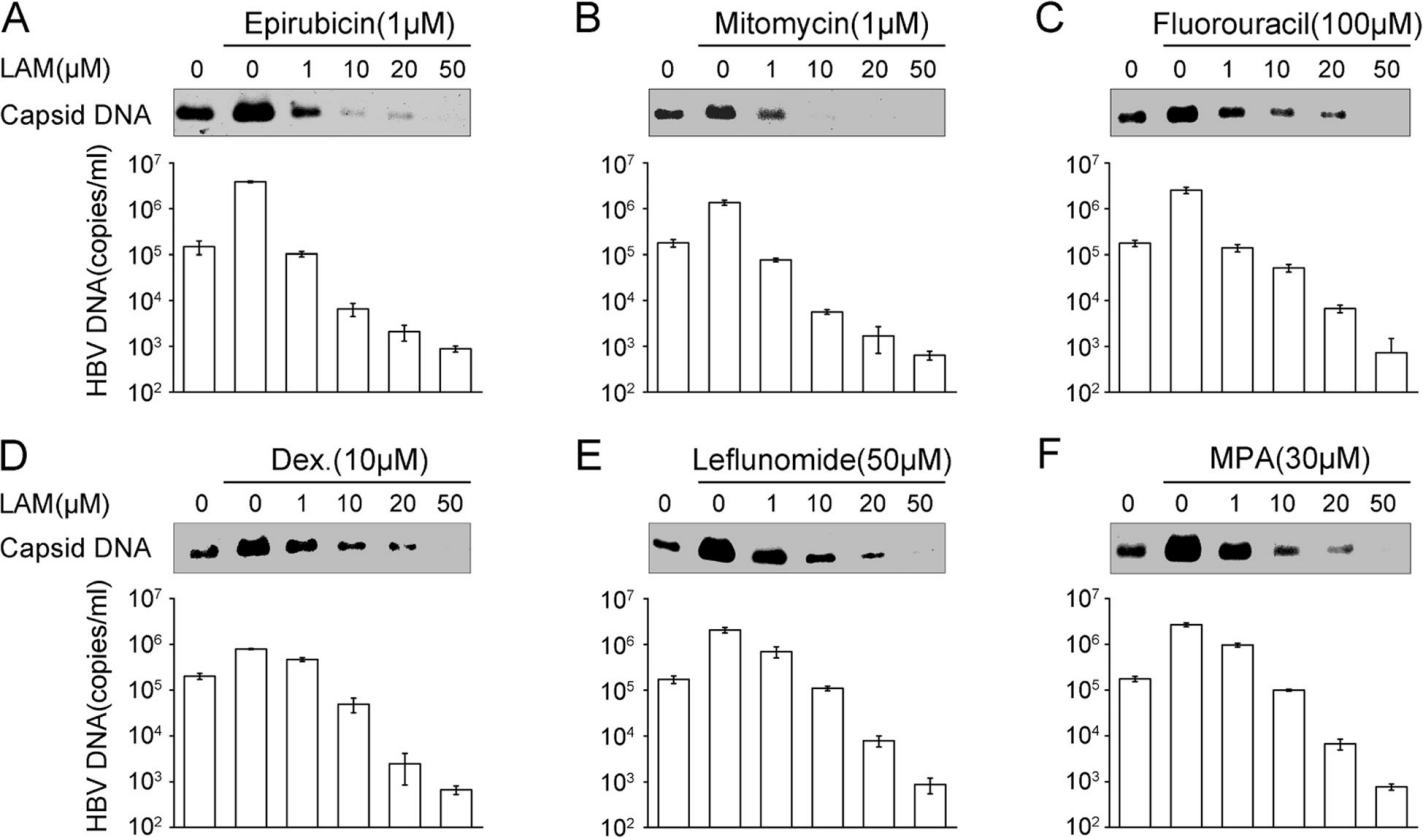
Lamivudine susceptibility assay. Lamivudine inhibits the activation of HBV replication in HepG2.2.15 cells treated with epirubicin, mitomycin, 5-fluorouracil, dexamethasone, leflunomide, and mycophenolic acid. HepG2.2.15 cells were treated with increasing concentrations of lamivudine for 24 h and then treated with 1 μM epirubicin (**a**), 1 μM mitomycin (**b**), or 100 μM 5-fluorouracil (**c**), for 16 h, or 10 μM dexamethasone (**d**), 50 μM leflunomide (**e**), or 30 μM mycophenolic acid (**f**), for 5 days. After incubation with increasing concentrations of lamivudine for 24 h, the cells were cultured in fresh media without drugs. Subsequently, intracellular capsid DNA was detected by molecular hybridization with the α-^32^P-labeled HBV DNA probe after NAGE (top). The number of extracellular HBV DNA copies was measured by qPCR (bottom), and the mean values from three isodose treatments, including SD, were statistically significant (*p* < 0.05)

In this study, we observed the effect of lamivudine (LAM) on HBV replication induced by epirubicin, mitomycin, 5-fluorouracil, dexamethasone, leflunomide, and mycophenolic acid, the LAM EC50 value was determined in previous research [21]. Native Southern blotting showed that intracellular capsid DNA signals were stronger in drug-treated cells with the absence of LAM, while almost no capsid DNA signals were detectable at the 50 μM LAM concentration after treating with 1 μM epirubicin (Fig. 5a, upper), 1 μM mitomycin (Fig. 5b, upper), 100 μM 5-fluorouracil (Fig. 5c, upper), 10 μM dexamethasone (Fig. 5d, upper), 50 μM leflunomide (Fig. 5e, upper), or 30 μM mycophenolic acid (Fig. 5f, upper). In addition, untreated HepG2.2.15 cells produced approximately 10^5^ copies/ml of virions. However, the concentration of LAM up to 50 μM decreased HBV copy numbers to the lower limit of detection compared with the HBV level above 10^6^ copies/ml in 1 μM epirubicin (Fig. 5a, bottom), 1 μM mitomycin (Fig. 5b, bottom), 100 μM 5-fluorouracil (Fig. 5c, bottom), 10 μM dexamethasone (Fig. 5d, bottom), 50 μM leflunomide (Fig. 5e, bottom) and 30 μM mycophenolic acid (Fig. 5f, bottom). These results confirmed that epirubicin, mitomycin, 5-fluorouracil, dexamethasone, leflunomide, and mycophenolic acid could enhance HBV DNA replication; however, this activation effect could not lower lamivudine sensitivity.

## Discussion

Reactivation of hepatitis B is a common complication in HBV infection patients undergoing cytotoxic chemotherapy or immunosuppressive therapy for cancer [2, 4], autoimmune diseases [7, 25] and organ transplantation [26]. Several clinical studies have reported that chemotherapy or immunosuppressive therapy, including epirubicin [16, 27, 28], mitomycin [1], 5-fluorouracil [1, 16], leflunomide [17], mycophenolic acid [18], and dexamethasone [13] treatment, could reactivate HBV replication.

Epirubicin, mitomycin, and 5-fluorouracil are currently used as cytotoxic chemotherapy drugs for cancer patients, such as those with hematological malignancies [29, 30], breast cancer [29, 30], digestive cancer [31], gastrointestinal cancer [32], and lung cancer [33]. In the clinic, cancer patients undergoing chemotherapy, including mitomycin treatment [1] or 5-fluorouracil treatment [1, 16], are at an increased risk of HBV reactivation. The effects of epirubicin on enhancing HBV replication have been reported [14]; however, the effects of mitomycin and 5-fluorouracil on HBV replication have not been investigated. HepG2.2.15 cells [19] were widely used to research HBV replication and expression because these cells can stably secrete HBV viral particles. HBV-NLuc-35 cells [20], which stem from HepG2 TA-7 cells transfected with the pTRE-sNLuc vector. In terms of pTRE-sNLuc vector, the secNLuc reporter gene binds downstream of the HBV C open reading frame (ORF) and upstream of the HBV P ORF. More importantly, the transcription product of the secNLuc gene is fused to recombinant HBV pregenomic RNA (pgRNA), which can be used to detect the expression and replication of HBV genes. By NAGE and Native Southern blotting, we observed that mitomycin and 5-fluorouracil could directly induce HBV replication and expression in vitro, rather than indirectly by affecting immunosuppression. This phenomenon was further confirmed by HBeAg ELISA, luciferase assay, and qPCR results. Different from epirubicin and 5-fluorouracil in concentration-dependent manner, when the concentration of mitomycin increased to 5 μM, the capsid signals from HBV-NLuc-35 cells was stronger compared with the weaker signals from HepG2.2.15 cells. By comparing HepG2.2.15 with HBV-Nluc-35 cells for their cell culturing status under different concentrations of Mitomycin treatment, the LD_50_ of Mitomycin in HepG2.2.15 cells was below HBV-Nluc-35, it was indicated that HepG2.2.15 cells were more sensitive to toxicity of Mitomycin than HBV-Nluc-35, consequently, producing different results in both cells. Previous study confirmed that mitomycin can strikingly activated HepG2 cells apoptosis [34]. Thus, we think that the type of HepG2.2.15 cells damage which mitomycin induced was apoptosis. In our syudy, in spite of plenty of HepG2.2.15 cells apoptosis, the level of HBV expression and replication of residual cells was the same as mitomycin-free according to HBeAg ELISA and qPCR results, supported that mitomycin can induce HBV replication and expression in concentration-dependent manner.

Leflunomide, mycophenolic acid and dexamethasone were regarded as immunosuppressants that could be used as therapy for autoimmune diseases, such as rheumatoid arthritis [35] and acute rejection of transplanted organs [36]. There was a report that dexamethasone could trigger the severity of hepatitis B virus-related chronic hepatitis in humans and could directly stimulate hepatitis B virus gene replication in vitro [13]. Interestingly, leflunomide has been reported to inhibit the replication of several viruses, such as human polyomavirus type [37, 38], human cytomegalovirus [39], herpes simplex virus type-1 [40], human immunodeficiency virus-1 [41] and respiratory syncytial virus [42], showing broad antiviral activities. However, in the clinic, there is a high risk of hepatitis B reactivation in rheumatoid arthritis patients with HBV infection after receiving leflunomide treatment [17]. Additionally, our study confirmed that leflunomide could significantly stimulate HBV gene expression and replication in vitro. This effect was not only observed in HepG2.2.15 cells but also in HBV-NLuc-35 cells. Mycophenolic acid is a highly effective immunosuppressant that is widely used to treat the rejection of solid organ transplants. As such, several reports have indicated that mycophenolic acid has antiviral activities against dengue virus [43], avian reovirus [44], yellow fever virus [45], and West Nile virus [46], especially hepatitis C virus [47–49] and hepatitis E virus [50]. However, a few studies have reported that the effect of mycophenolic acid on HBV replication ranges from weak suppression to no effect [51, 52]. In this study, we clearly demonstrated that mycophenolic acid could enhance HBV gene expression and replication in vitro, rather than have no effect or antiviral activities.

There were several published researches attempting to explore the molecular mechanism of hepatitis B virus reactivation. Hsu CH suspected that cytotoxic chemotherapy drug induced HBV reactivation due to interfere the normal cell cycle process and block at G2/M –phase [15]. Chen YF demonstrated that cytotoxic chemotherapy drug elevated the expression of the cell cycle regulator p21 (Waf1/Cip1) and CCAAT/enhancer-binding protein α (C/EBPα) contributed to the reactivation of HBV [14], which could interactive with responsive sites on the HBV promoters and effect the transcription of HBV pgRNA. Mouler Rechtman M indicated that peroxisome proliferator-activated receptor-gamma coactivator-1α (PGC-1α) play a central mediator in HBV reactivation induced by cytotoxic chemotherapy drugs, which strongly coactivate for HBV transcription [53]. Huang W showed that immunosuppressant could activate cellular autophagy to induce HBV reactivation [54]. Hoppe-Seyler K proved that immunosuppressant induced HBV reactivation was closely linked with increasing of mitogen-activated protein kinase p38 phosphorylation, which was crucial for pgRNA increasing [55]. HBV pgRNA plays an important role in the HBV life cycle, it serves as mRNA encoding the core protein (HBcAg) and the viral reverse transcriptase, moreover, it can be packaged into progeny capsids and converted into relaxed circular DNA (RC-DNA) [56]. A new explanation for HBV reactivation is that accumulation of HBV Pol promotes the activity of the viral polymerase [57]. Thus, we speculate that different drugs might have different molecular mechanisms of HBV reactivation, pgRNA might play an important role in this procedure.

The reactivation of hepatitis B occurs not only in overt HBV infected patients who are HBV surface antigen positive (HBsAg+) and receive cytotoxic chemotherapy or immunosuppressive therapy [26] but also in patients with resolved infection that are HBV surface antigen negative (HBsAg-), HBV core antibody positive (HBcAb+), and positive or negative for antibodies against HBV surface antigen (HBsAb+) [58, 59]; consequently, we should pay close attention to this phenomenon in the clinic. Several studies have reported that HBV could maintain a lower level of replication ability in both hepatocytes and peripheral blood mononuclear cells in resolved HBV infect patients [60, 61] and that cccDNA is very difficult to eliminate, even though when undetectable in the serum. This is an explanation why HBV reactivation can occur in patients with resolved HBV infection.

In this study, we observed that LAM could counteractive the reactivation of HBV replication induced by epirubicin, mitomycin, 5-fluorouracil, leflunomide, mycophenolic acid and dexamethasone in vitro. LAM (nucleoside analog, NA), as a well-characterized antiviral drug, is used to treat chronic HBV infection [62, 63]. Thus, in the clinic, it is necessary that patients with overt HBV infection receive antiviral therapy before receiving cytotoxic chemotherapy or immunosuppressive therapy [10]. Most importantly, HBV reactivation can occur in patients with resolved HBV infection as well; however, the risk is lower [64]. At present, the controversy continues, and it is not unequivocally agreed whether prophylactic antiviral therapy should be prescribed to patients who are HBsAg negative and HBcAb positive with or without HBsAb positivity. Given our research, cytotoxic chemotherapy drugs and immunosuppressants could directly stimulate the replication and expression of HBV rather than indirectly through immunosuppression. And, given that cccDNA is difficult to eliminate, closely monitoring changes in HBV serological markers to prevent HBV reactivation is necessary when patients with resolved HBV infection receive cytotoxic chemotherapy or immunosuppressive therapy. Hence, screening the most sensitive serological marker is important for monitoring HBV reactivation, and based on our research, HBV pgRNA may be a promising candidate.

## Conclusions

In our study, we confirmed that mitomycin, 5-fluorouracil, leflunomide and mycophenolic acid can directly upregulated HBV replication and expression in vitro rather than indirectly through immunosuppression. Moreover, this effect could be prevented by nucleoside analogs, such as lamivudine (LAM). Thus, HBV reactivation is a serious but avoidable complication of receiving cytotoxic chemotherapy or immunosuppressive therapy and could be prevented with the use of prophylactic antiviral therapy.

## Supplementary information

**Additional file 1: Figure S1.** Cell proliferation toxicity test of HepG2.2.15 and HBV-Nluc-35 cells treated with the different concentrations of Mitomycin. For comparing HepG2.2.15 (bottom) with HBV-Nluc-35 (top) cells for their cell culturing status under different concentrations of Mitomycin, both cells were treated with increasing concentrations of mitomycin for 16 h. Cell proliferation toxicity was measured using CCK-8 reagent as recommended by the manufacturer (Dojindo). For HBV-Nluc-35 cells, the LD_50_ of Mitomycin was 8.562 μM, and the 95% CI of the LD_50_ was 5.71–34.98 μM. It was indicated that HepG2.2.15 cells were more sensitive to toxicity of Mitomycin than HBV-Nluc-35.

## Data Availability

Not applicable.

## Abbreviations

HBV: Hepatitis B virus
HBsAg: HBV surface antigen
HBcAb: HBV core antibody
HBeAg: HBV e antigen
RC-DNA: Relaxed circular DNA
cccDNA: Covalently closed circular DNA
pgRNA: Pregenomic RNA
ORF: Open reading frame
LAM: Lamivudine
NAGE: Native agarose gel electrophoresis
qPCR): Quantitative PCR
ELISA: Enzyme-linked immunosorbent assay
LD_50_: Median lethal dose
EC50: 50% effective concentration

## References

[1] Wijaya I, Hasan I (2013). Reactivation of hepatitis B virus associated with chemotherapy and immunosuppressive agent. Acta Med Indones.

[2] Cheung KS, Seto WK, Lai CL, Yuen MF. Prevention and management of hepatitis B virus reactivation in cancer patients. Hepatol Int. 2016 [PMID: 26739135. 10.1007/s12072-015-9692-3. 10.1007/s12072-015-9692-3 26739135

[3] Lo Re V, Schuster M (2016). Evaluating hepatitis B virus reactivation during solid tumor chemotherapy: evidence to guide pretreatment hepatitis B screening and prophylaxis. Ann Intern Med.

[4] Paul S, Saxena A, Terrin N, Viveiros K, Balk EM, Wong JB (2016). Hepatitis B virus reactivation and prophylaxis during solid tumor chemotherapy: a systematic review and meta-analysis. Ann Intern Med.

[5] Wands JR, Chura CM, Roll FJ, Maddrey WC (1975). Serial studies of hepatitis-associated antigen and antibody in patients receiving antitumor chemotherapy for myeloproliferative and lymphoproliferative disorders. Gastroenterology.

[6] Perrillo RP (2001). Acute flares in chronic hepatitis B: the natural and unnatural history of an immunologically mediated liver disease. Gastroenterology.

[7] Hwang JP, Lok AS (2014). Management of patients with hepatitis B who require immunosuppressive therapy. Nat Rev Gastroenterol Hepatol.

[8] Ikeda M (2013). Reactivation of hepatitis B virus in patients receiving chemotherapy. Jpn J Clin Oncol.

[9] Liu CJ, Chen PJ, Chen DS, Kao JH (2013). Hepatitis B virus reactivation in patients receiving cancer chemotherapy: natural history, pathogenesis, and management. Hepatol Int.

[10] Oketani M, Ido A, Uto H, Tsubouchi H (2012). Prevention of hepatitis B virus reactivation in patients receiving immunosuppressive therapy or chemotherapy. Hepatol Res.

[11] Di Bisceglie AM, Lok AS, Martin P, Terrault N, Perrillo RP, Hoofnagle JH (2015). Recent US Food and Drug Administration warnings on hepatitis B reactivation with immune-suppressing and anticancer drugs: just the tip of the iceberg?. Hepatology.

[12] Hoofnagle JH (2009). Reactivation of hepatitis B. Hepatology.

[13] Tur-Kaspa R, Laub O (1990). Corticosteroids stimulate hepatitis B virus DNA, mRNA and protein production in a stable expression system. J Hepatol.

[14] Chen YF, Chong CL, Wu YC, Wang YL, Tsai KN, Kuo TM, Hong MH, Hu CP, Chen ML, Chou YC, Chang C (2015). Doxorubicin Activates Hepatitis B Virus Replication by Elevation of p21 (Waf1/Cip1) and C/EBPalpha Expression. PloS one.

[15] Hsu CH, Hsu HC, Chen HL, Gao M, Yeh PY, Chen PJ, Cheng AL (2004). Doxorubicin activates hepatitis B virus (HBV) replication in HBV-harboring hepatoblastoma cells. A possible novel mechanism of HBV reactivation in HBV carriers receiving systemic chemotherapy. Anticancer Res.

[16] Yeo W, Lam KC, Zee B, Chan PS, Mo FK, Ho WM, Wong WL, Leung TW, Chan AT, Ma B, Mok TS, Johnson PJ (2004). Hepatitis B reactivation in patients with hepatocellular carcinoma undergoing systemic chemotherapy. Ann Oncol.

[17] Ming-Xu H, Chen M, Cai Y, Yan-Jia H (2015). Clinical outcomes of low-dose leflunomide for rheumatoid arthritis complicated with Hepatitis B virus carriage and safety observation. Pak J Med Sci.

[18] Sayarlioglu H, Erkoc R, Dogan E, Sayarlioglu M, Topal C (2005). Mycophenolate mofetil use in hepatitis B associated-membranous and membranoproliferative glomerulonephritis induces viral replication. Ann Pharmacother.

[19] Sells MA, Chen ML, Acs G (1987). Production of hepatitis B virus particles in Hep G2 cells transfected with cloned hepatitis B virus DNA. Proc Nat Acad Sci U S A.

[20] Ruan J, Ping CY, Sun S, Cheng X, Han PY, Zhang YG, Sun DX (2019). Construction of a replication-competent hepatitis B virus vector carrying secreted luciferase transgene and establishment of new hepatitis B virus replication and expression cell lines. World J Gastroenterol.

[21] Sun D, Nassal M (2006). Stable HepG2- and Huh7-based human hepatoma cell lines for efficient regulated expression of infectious hepatitis B virus. J Hepatol.

[22] Wang Z, Wu L, Cheng X, Liu S, Li B, Li H, Kang F, Wang J, Xia H, Ping C, Nassal M, Sun D (2013). Replication-competent infectious hepatitis B virus vectors carrying substantially sized transgenes by redesigned viral polymerase translation. PloS one.

[23] Birnbaum F, Nassal M (1990). Hepatitis B virus nucleocapsid assembly: primary structure requirements in the core protein. J Virol.

[24] Ren S, Nassal M (2001). Hepatitis B virus (HBV) virion and covalently closed circular DNA formation in primary tupaia hepatocytes and human hepatoma cell lines upon HBV genome transduction with replication-defective adenovirus vectors. J Virol.

[25] Kato M, Atsumi T, Kurita T, Odani T, Fujieda Y, Otomo K, Horita T, Yasuda S, Koike T (2011). Hepatitis B virus reactivation by immunosuppressive therapy in patients with autoimmune diseases: risk analysis in Hepatitis B surface antigen-negative cases. J Rheumatol.

[26] Manzano-Alonso ML, Castellano-Tortajada G (2011). Reactivation of hepatitis B virus infection after cytotoxic chemotherapy or immunosuppressive therapy. World J Gastroenterol.

[27] Koo YX, Tay M, Teh YE, Teng D, Tan DS, Tan IB, Tai DW, Quek R, Tao M, Lim ST (2011). Risk of hepatitis B virus (HBV) reactivation in hepatitis B surface antigen negative/hepatitis B core antibody positive patients receiving rituximab-containing combination chemotherapy without routine antiviral prophylaxis. Ann Hematol.

[28] Yeo W, Chan PK, Zhong S, Ho WM, Steinberg JL, Tam JS, Hui P, Leung NW, Zee B, Johnson PJ (2000). Frequency of hepatitis B virus reactivation in cancer patients undergoing cytotoxic chemotherapy: a prospective study of 626 patients with identification of risk factors. J Med Virol.

[29] Kumagai K, Takagi T, Nakamura S, Sawada U, Kura Y, Kodama F, Shimano S, Kudoh I, Nakamura H, Sawada K, Ohnoshi T (1997). Hepatitis B virus carriers in the treatment of malignant lymphoma: an epidemiological study in Japan. Ann Oncol.

[30] Nakamura Y, Motokura T, Fujita A, Yamashita T, Ogata E (1996). Severe hepatitis related to chemotherapy in hepatitis B virus carriers with hematologic malignancies. Survey in Japan, 1987-1991. Cancer.

[31] Liaw YF (1998). Hepatitis viruses under immunosuppressive agents. J Gastroenterol Hepatol.

[32] Yang Y, Du Y, Luo WX, Li C, Chen Y, Cheng K, Ding J, Zhou Y, Ge J, Yang X, Liu JY (2015). Hepatitis B virus reactivation and hepatitis in gastrointestinal cancer patients after chemotherapy. Cancer Chemother Pharmacol.

[33] Lin GN, Peng JW, Xiao JJ, Liu DY, Xia ZJ (2014). Hepatitis B virus reactivation in hepatitis B surface antigen seropositive patients with metastatic non-small cell lung cancer receiving cytotoxic chemotherapy: the efficacy of preemptive lamivudine and identification of risk factors. Med Oncol.

[34] Castaneda F, Kinne RK (1999). Effects of doxorubicin, mitomycin C, and ethanol on Hep-G2 cells in vitro. J Cancer Res Clin Oncol.

[35] Teschner S, Burst V (2010). Leflunomide: a drug with a potential beyond rheumatology. Immunotherapy.

[36] Allison AC (2005). Mechanisms of action of mycophenolate mofetil. Lupus.

[37] Jeffers-Francis LK, Burger-Calderon R, Webster-Cyriaque J (2015). Effect of Leflunomide, Cidofovir and Ciprofloxacin on replication of BKPyV in a salivary gland in vitro culture system. Antiviral Res.

[38] Farasati NA, Shapiro R, Vats A, Randhawa P (2005). Effect of leflunomide and cidofovir on replication of BK virus in an in vitro culture system. Transplantation.

[39] Waldman WJ, Knight DA, Blinder L, Shen J, Lurain NS, Miller DM, Sedmak DD, Williams JW, Chong AS (1999). Inhibition of cytomegalovirus in vitro and in vivo by the experimental immunosuppressive agent leflunomide. Intervirology.

[40] Knight DA, Hejmanowski AQ, Dierksheide JE, Williams JW, Chong AS, Waldman WJ (2001). Inhibition of herpes simplex virus type 1 by the experimental immunosuppressive agent leflunomide. Transplantation.

[41] Schlapfer E, Fischer M, Ott P, Speck RF (2003). Anti-HIV-1 activity of leflunomide: a comparison with mycophenolic acid and hydroxyurea. AIDS.

[42] Dunn MC, Knight DA, Waldman WJ (2011). Inhibition of respiratory syncytial virus in vitro and in vivo by the immunosuppressive agent leflunomide. Antiviral Ther.

[43] Diamond MS, Zachariah M, Harris E (2002). Mycophenolic acid inhibits dengue virus infection by preventing replication of viral RNA. Virology.

[44] Robertson CM, Hermann LL, Coombs KM (2004). Mycophenolic acid inhibits avian reovirus replication. Antiviral Res.

[45] Leyssen P, Balzarini J, De Clercq E, Neyts J (2005). The predominant mechanism by which ribavirin exerts its antiviral activity in vitro against flaviviruses and paramyxoviruses is mediated by inhibition of IMP dehydrogenase. J Virol.

[46] Morrey JD, Smee DF, Sidwell RW, Tseng C (2002). Identification of active antiviral compounds against a New York isolate of West Nile virus. Antivir Res.

[47] Chen H, Ye L, Su JM, Li Y, Zeng JR, Huo WZ (2013). Inhibition of hepatitis C virus replication by mycophenolic acid in hepatocytes. Zhonghua shi yan he lin chuang bing du xue za zhi = Zhonghua shiyan he linchuang bingduxue zazhi = Chinese journal of experimental and clinical virology.

[48] Pan Q, de Ruiter PE, Metselaar HJ, Kwekkeboom J, de Jonge J, Tilanus HW, Janssen HL, van der Laan LJ (2012). Mycophenolic acid augments interferon-stimulated gene expression and inhibits hepatitis C Virus infection in vitro and in vivo. Hepatology.

[49] Henry SD, Metselaar HJ, Lonsdale RC, Kok A, Haagmans BL, Tilanus HW, van der Laan LJ (2006). Mycophenolic acid inhibits hepatitis C virus replication and acts in synergy with cyclosporin A and interferon-alpha. Gastroenterology.

[50] Wang Y, Zhou X, Debing Y, Chen K, Van Der Laan LJ, Neyts J, Janssen HL, Metselaar HJ, Peppelenbosch MP, Pan Q (2014). Calcineurin inhibitors stimulate and mycophenolic acid inhibits replication of hepatitis E virus. Gastroenterology.

[51] Gong ZJ, De Meyer S, Clarysse C, Verslype C, Neyts J, De Clercq E, Yap SH (1999). Mycophenolic acid, an immunosuppressive agent, inhibits HBV replication in vitro. J Viral Hepat.

[52] Wu J, Xie HY, Jiang GP, Xu X, Zheng SS (2003). The effect of mycophenolate acid on hepatitis B virus replication in vitro. Hepatobiliary Pancreat Dis Int.

[53] Mouler Rechtman M, Burdelova EO, Bar-Yishay I, Ben-Yehoyada M, Fishman S, Halpern Z, Shlomai A (2013). The metabolic regulator PGC-1alpha links anti-cancer cytotoxic chemotherapy to reactivation of hepatitis B virus. J Viral Hepatitis.

[54] Huang W, Zhao F, Huang Y, Li X, Zhu S, Hu Q, Chen W (2014). Rapamycin enhances HBV production by inducing cellular autophagy. Hepatitis Monthly.

[55] Hoppe-Seyler K, Sauer P, Lohrey C, Hoppe-Seyler F (2012). The inhibitors of nucleotide biosynthesis leflunomide, FK778, and mycophenolic acid activate hepatitis B virus replication in vitro. Hepatology.

[56] Ou JH, Bao H, Shih C, Tahara SM (1990). Preferred translation of human hepatitis B virus polymerase from core protein- but not from precore protein-specific transcript. J Virol.

[57] Hou L, Zhao J, Gao S, Ji T, Song T, Li Y, Wang J, Geng C, Long M, Chen J, Lin H, Cai X, Cang Y (2019). Restriction of hepatitis B virus replication by c-Abl-induced proteasomal degradation of the viral polymerase. Sci Advances.

[58] Koo YX, Tan DS, Tan BH, Quek R, Tao M, Lim ST (2009). Risk of hepatitis B virus reactivation in patients who are hepatitis B surface antigen negative/antibody to hepatitis B core antigen positive and the role of routine antiviral prophylaxis. J Clin Oncol.

[59] Masarone M, De Renzo A, La Mura V, Sasso FC, Romano M, Signoriello G, Rosato V, Perna F, Pane F, Persico M (2014). Management of the HBV reactivation in isolated HBcAb positive patients affected with Non Hodgkin Lymphoma. BMC Gastroenterol.

[60] Rehermann B, Ferrari C, Pasquinelli C, Chisari FV (1996). The hepatitis B virus persists for decades after patients' recovery from acute viral hepatitis despite active maintenance of a cytotoxic T-lymphocyte response. Nat Med.

[61] Catterall AP, Murray-Lyon IM, Zuckerman AJ, Harrison TJ (1994). Southern hybridisation analysis of HBV DNA in peripheral blood leucocytes and of different cell types: changes during the natural history and with interferon-alpha therapy in patients with hepatitis B virus infection. J Med Virol.

[62] Lisotti A, Azzaroli F, Buonfiglioli F, Montagnani M, Alessandrelli F, Mazzella G (2008). Lamivudine treatment for severe acute HBV hepatitis. Int J Med Sci.

[63] Cheng X, Guan W, Sun S, Li B, Li H, Kang F, Kang J, Yang D, Nassal M, Sun D (2015). Stable human hepatoma cell lines for efficient regulated expression of nucleoside/nucleotide analog resistant and vaccine escape hepatitis B virus variants and woolly monkey hepatitis B virus. PloS one.

[64] Morisco F, Guarino M, La Bella S, Di Costanzo L, Caporaso N, Ayala F, Balato N (2014). Lack of evidence of viral reactivation in HBsAg-negative HBcAb-positive and HCV patients undergoing immunosuppressive therapy for psoriasis. BMC Gastroenterol.

