# Supplementary material for: Mitomycin, 5-fluorouracil, leflunomide, and mycophenolic acid directly promote hepatitis B virus replication and expression in vitro
Source: Virol J. 2020 Jul 1;17:89. doi: 10.1186/s12985-020-01339-5 (PMC7331192; doi:10.1186/s12985-020-01339-5)
Supplement: Supplementary file 1 — Additional file 1: Figure S1. Cell proliferation toxicity test of HepG2.2.15 and HBV-Nluc-35 cells treated with the different concentrations of Mitomycin. For comparing HepG2.2.15 (bottom) with HBV-Nluc-35 (top) cells for their cell culturing status under different concentrations of Mitomycin, both cells were treated with increasing concentrations of mitomycin for 16 h. Cell proliferation toxicity was measured using CCK-8 reagent as recommended by the manufacturer (Dojindo). For HBV-Nluc-35 cells, the LD50 of Mitomycin was 8.562 μM, and the 95% CI of the LD50 was 5.71–34.98 μM. It was indicated that HepG2.2.15 cells were more sensitive to toxicity of Mitomycin than HBV-Nluc-35. [file 12985_2020_1339_MOESM1_ESM.doc]

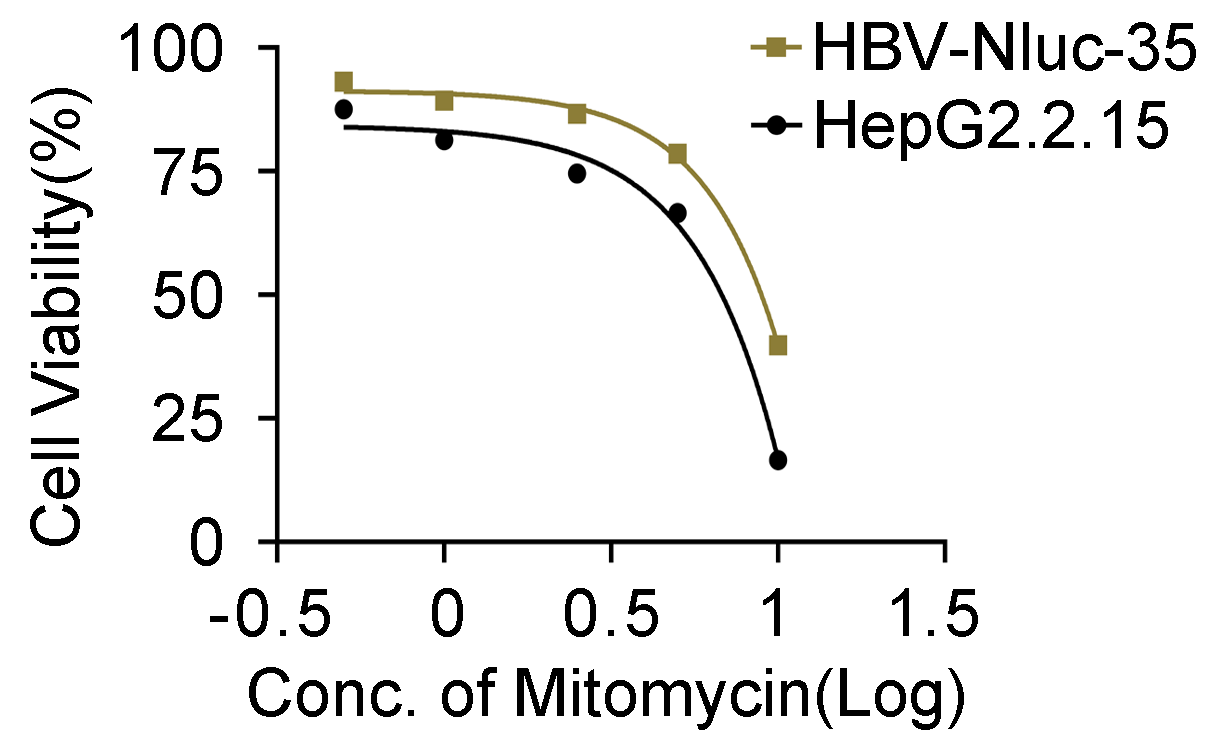


***Supplementary Fig. Cell proliferation toxicity test of HepG2.2.15 and HBV-Nluc-35 cells treated with the different concentrations of Mitomycin.*** *For comparing HepG2.2.15 (bottom) with HBV-Nluc-35 (top) cells for their cell culturing status under different concentrations of Mitomycin, both cells were treated with increasing concentrations of mitomycin for 16 hours. Cell proliferation toxicity was measured using CCK-8 reagent as recommended by the manufacturer (Dojindo). For HBV-Nluc-35 cells, the LD_50_ of Mitomycin was 8.562 µM, and the 95% CI of the LD_50_ was 5.71-34.98 µM. It was indicated that HepG2.2.15 cells were more sensitive to toxicity of Mitomycin than HBV-Nluc-35.*
